# Modeling the effect of different drugs and treatment regimen for hookworm on cure and egg reduction rates taking into account diagnostic error

**DOI:** 10.1371/journal.pntd.0010810

**Published:** 2022-10-04

**Authors:** Carla M. Grolimund, Oliver Bärenbold, Jürg Utzinger, Jennifer Keiser, Penelope Vounatsou

**Affiliations:** 1 Swiss Tropical and Public Health Institute, Allschwil, Switzerland; 2 University of Basel, Basel, Switzerland; Royal Veterinary College, UNITED KINGDOM

## Abstract

**Background:**

Hookworm infections, caused by *Ancylostoma duodenale* and *Necator americanus*, are of considerable public health importance. The World Health Organization recommends preventive chemotherapy as the key strategy for morbidity control. Meta-analyses have been conducted to estimate treatment efficacy of available drugs and drug combinations. However, in most studies, the relation between the diagnostic error and infection intensity have not been considered, resulting in an overestimation of cure rates (CRs).

**Methodology:**

A Bayesian model was developed to compare the ‘true’ CR and egg reduction rate of different treatment regimens for hookworm infections taking into account the error of the recommended Kato-Katz thick smear diagnostic technique. It was fitted to the observed egg count data which was linked to the distribution of worms, considered the day-to-day variation of hookworm egg excretion and estimated the infection intensity-dependent sensitivity. The CR was obtained by defining the prevalence of infection at follow-up as the probability of having at least one fertilized female worm. The model was applied to individual-level egg count data available from 17 treatments and six clinical trials.

**Principal findings:**

Taking the diagnostic error into account resulted in considerably lower CRs than previously reported. Overall, of all treatments analyzed, mebendazole administered in six dosages of 100 mg each was the most efficacious treatment with a CR of 88% (95% Bayesian credible interval: 79-95%). Furthermore, diagnostic sensitivity varied with the infection intensity and sampling effort. For an infection intensity of 50 eggs per gram of stool, the sensitivity is close to 60%; for two Kato-Katz thick smears it increased to approximately 76%.

**Conclusions/significance:**

Our model-based estimates provide the true efficacy of different treatment regimens against hookworm infection taking into account the diagnostic error of the Kato-Katz method. Estimates of the diagnostic sensitivity for different number of stool samples and thick smears are obtained. To accurately assess efficacy in clinical trials with the Kato-Katz method, at least two stool samples on consecutive days should be collected.

## Author summary

Human hookworm infections are primarily caused by two parasitic worm species, namely *Ancylostoma duodenale* and *Necator americanus*. Already moderate infection intensities can impair cognitive and physical development of children, and reduce fertility in women of reproductive age. The World Health Organization set the global target to eliminate morbidity due to hookworm infections in preschool-age and school-age children by 2030. To reach this goal, different public health interventions are carried out. The most widely used strategy is mass drug administration. To assess the efficacy of treatments, epidemiologic studies and clinical trials usually employ the Kato-Katz thick smear technique to microscopically examine stool samples of individuals before and after treatment. Of note, this diagnostic test is not 100% accurate such that not all positive individuals are detected. We developed a mathematical model, which takes into account the diagnostic error to compare the ‘true’ efficacy of different drugs and treatment regimens against hookworm infection. Furthermore, we computed how well the test detects infected individuals in relation to infection intensity. We found that the diagnostic sensitivity of the Kato-Katz thick smear technique increases considerably if two stool specimens from different days are collected and multiple Kato-Katz thick smears are analyzed instead of one.

## Introduction

Human hookworm infections are primarily caused by *Ancylostoma duodenale* and *Necator americanus* [1]. It is estimated that around 450 million people are infected with hookworm and an equivalent of roughly 1.685 million years are lost due to disability annually [2]. Impaired cognitive and physical development in children, as well as reduced fertility among women of reproductive age due to iron deficiency and anemia are important morbidities caused by moderate or heavy hookworm infection [3]. Elimination of morbidity due to hookworm and other soil-transmitted helminth (STH) species in preschool-age and school-age children by 2030 is the global target set forth by the World Health Organization (WHO) [4]. To reduce the burden caused by hookworm and other STH species, different control measures are being implemented, such as footwear campaigns, water, sanitation, and hygiene (WASH) interventions, and preventive chemotherapy (PC) that is the periodic administration of anthelmintic drugs without prior diagnosis [5]. PC is the most widely used intervention and has been shown to reduce the burden especially of moderate and heavy STH infection intensities [5–7].

WHO recommends single doses of 400 mg albendazole and 500 mg mebendazole in PC campaigns against hookworm and other STH infections [1]. Recent studies and meta-analyses have shown that other regimens and combination treatment are more efficacious. For instance, Palmeirim et al. (2018) found that six dosages of 100 mg mebendazole are more efficacious against hookworm infections than a single dose of 500 mg [8]. Moser et al. (2018) presented data from a trial conducted in Lao People’s Democratic Republic (Lao PDR) highlighting high efficacy of a combination of albendazole, pyrantel pamoate plus oxantel pamoate against hookworm infections [9].

Systematic reviews and meta-analyses have been carried out to compare the efficacy of different treatments against hookworm and other STH infections [10–12]. However, most trials assess the presence of hookworms using the Kato-Katz thick smear technique, which has low sensitivity, and hence, the efficacy of the treatment is overestimated. Meta-analyses using latent class models (LCMs) have been conducted to take into account the diagnostic error [13–15]. It must be noted, however, that these models analyze only the positive/negative test result for each individual. In contrast, Bärenbold et al. (2017) developed an individual-level egg count Bayesian model, which takes into account the dependence of sensitivity on the infection intensity and includes the day-to-day variation in helminth egg output [16]. The model has been succesfully applied to data from a clinical trial on Pemba Island to assess the performance of FECPAK^G2^ and the Kato-Katz thick smear technique for the diagnosis of STH infection [17].

In this study, we pursued a Bayesian meta-analysis to compare the ‘true’ cure rate (CR) and egg reduction rate (ERR) of different treatments against hookworm infection considering the diagnostic error of the Kato-Katz thick smear technique. We fitted a model to the observed egg count data that took into account the distribution of worms and estimated diagnostic sensitivity as a function of the infection intensity. We considered day-to-day variation in the egg counts and correlation of slides from the same stool sample. The CR was estimated by defining the prevalence as the probability of having at least one fertilized female worm. Our analysis includes data from six clinical trials, which comprise 17 unique treatments based on a single drug or a combination of two or three anthelmintic drugs.

## Materials and methods

### Ethics statement

The studies from which the data used in this analysis were obtained were published elsewhere [9, 18–22]. Details on ethical approvals, trial registration, study design, informed consent procedures, potential risks and benefits are provided in the aforementioned studies.

### Data

We analyzed data from six randomized trials in Côte d’Ivoire, Lao PDR, and Tanzania, which assessed the efficacy and safety of different treatments against STH infection [9, 18–22]. All trials followed the same sampling design and used the Kato-Katz thick smear technique. For each individual, two stool specimens were collected over two consecutive days at baseline and treatment follow-up 14–21 days post-treatment, while two readings were made per specimen. All slides were read within 1 hour after preparation to avoid degeneration of hookworm eggs on microscope slides. At baseline, we only considered hookworm-positive individuals, and hence the prevalence was set at 100%. For trials with a focus on *T. trichiura*, only hookworm-positive individuals were included in the analysis. The observed CRs against hookworm ranged from 11% to 98% and the ERRs from 11% to 100%. A summary of the data, including the treatment arms by trial, is provided in Table 1. The confidence intervals (CIs) were calculated in R (version 3.6.1), using the package ‘prevalence’ for the CRs with Jeffreys method and ‘eggCounts’ for the ERRs with bootstrapping.

**Table 1:** Description of trial data: Age group included, treatments tested, sample sizes and arithmetic mean of averaged egg counts per gram of stool (EPG) averaged on the four slides at baseline (BL) and follow-up (FU).

Trial	Country	Age (years)	Treatment	No. of participants with hookworm infection	Mean EPG at BL	Mean EPG at FU
Coulibaly et al. (2017) [19]	Côte d’Ivoire	6–13	Tribendimidine 100 mg	33	355	258
			Tribendimidine 200 mg	31	313	130
			Tribendimidine 400 mg	31	215	96
Moser et al. (2016) [9]	Côte d’Ivoire	15–18	Tribendimidine 400 mg	55	421	106
			Tribendimidine 400 mg+ivermectin 200 μg/kg	54	485	27
			Tribendimidine 400 mg+oxantel pamoate 25 mg/kg	53	424	103
			Albendazole 400 mg+oxantel pamoate 25 mg/kg	49	629	113
Moser et al. (2016) [9]	Tanzania	6–14	Tribendimidine 400 mg	96	399	84
			Tribendimidine 400 mg+ivermectin 200 μg/kg	100	377	9
			Tribendimidine 400 mg+oxantel pamoate 25 mg/kg	95	385	57
			Albendazole 400 mg+oxantel pamoate 25 mg/kg	99	459	42
Moser et al. (2017) [18]	Lao PDR	6–12	Albendazole 400 mg+oxantel pamoate 20 mg/kg	138	1,269	114
			Pyrantel pamoate 20 mg/kg+oxantel pamoate 20 mg/kg	68	1,317	49
			Albendazole 400 mg+pyrantel pamoate 20 mg/kg+oxantel pamoate 20 mg/kg	138	1,374	22
			Mebendazole 500 mg+pyrantel pamoate 20 mg/kg+oxantel pamoate 20 mg/kg	69	1,457	99
Palmeirim et al. (2017) [22]	Tanzania	6–14	Mebendazole 500 mg	92	443	209
			Mebendazole 6x100 mg	93	465	1
Speich et al. (2012) [20]	Tanzania	15–18	Albendazole 400 mg+oxantel pamoate 20 mg/kg^1^	109	434	56
			Oxantel pamoate 20 mg/kg	113	276	238
			Albendazole 400 mg	112	236	57
			Mebendazole 500 mg	108	312	173
Speich et al. (2013) [21]	Tanzania	6–15	Albendazole 400 mg+ivermectin 200 μg/kg	42	338	35
			Albendazole 400 mg+mebendazole 500 mg	46	388	106
			Albendazole 400 mg+oxantel pamoate 20 mg/kg	55	222	66
			Mebendazole 500 mg	41	173	153

^1^ Administered on 2 consecutive days

### Model

**Fig 1:**
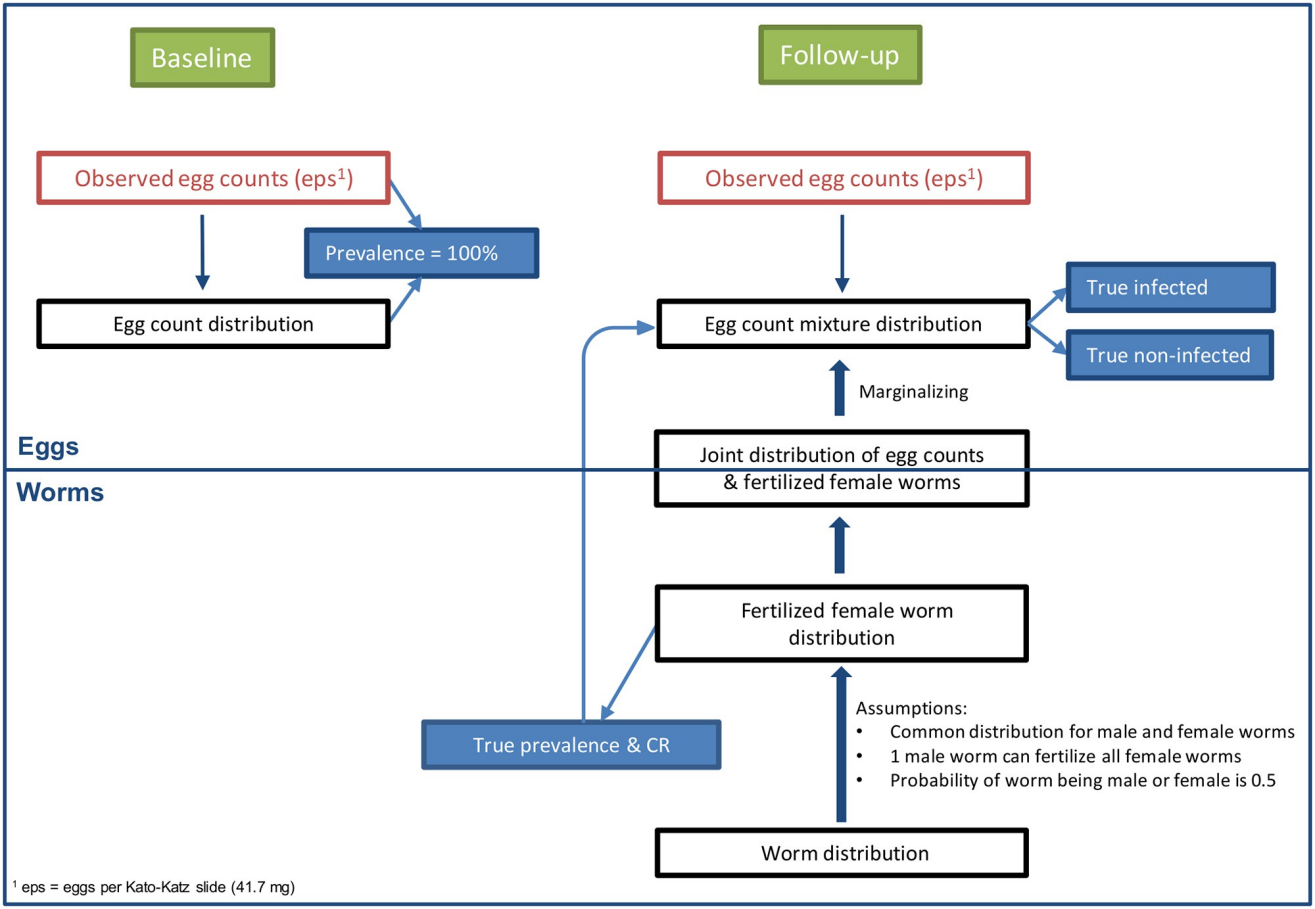
Graphical representation of the model. Numbers in brackets indicate equation numbers as given in the text below and in S2 Appendix.

We developed a model, fitted to the individual egg count data assuming they arise from a negative binomial distribution at baseline and a mixture of negative binomial distributions at follow-up with mixing proportion equal to the ‘true’ prevalence of infection in the population. The latter was defined as the probability of having at least one fertilized female worm and derived from a negative binomial distribution of worms, assuming a 1:1 female to male ratio and that one male worm can fertilize all females. We considered conditional independence of the slides given the disease status to take into account the correlation of slides from the same stool sample and took into account day-to-day variation on the individual egg counts. We estimated the ‘true’ CR from the ‘true’ prevalence at follow-up. To link the egg counts to the worm burden, we computed the marginal distribution of egg counts from the joint distribution of egg counts and fertilized female worms. The density dependence was not taken into account due to the low infection intensities at follow-up. The ERR was calculated from the treatment group specific mean egg burden at baseline and at follow-up (among the truly infected individuals). The model formulation enabled estimation of the egg intensity-dependent sensitivity of the Kato-Katz method for different number of slides. A mathematical description of the model and a graphical representation are given below, in Table 2 and Fig 1, respectively.

**Table 2:** Description of parameters used in the model.

	Parameter	Description
Eggs	$\mu_{i_{j\;g}d}^{\left(t\right)}$	Daily individual mean egg burden at baseline (*t* = 0) and follow-up (*t* = 1)
	$\mu_{i_{j\;g}}^{\left(t\right)}$	Individual mean egg burden at baseline (*t* = 0) and follow-up (*t* = 1)
	$\mu_{j\;g}^{\left(t\right)}$	Mean egg burden of treatment group *g* from survey *j* at baseline (*t* = 0) and follow-up (*t* = 1)
	*k* ^(*t*)^	Aggregation in infected individuals at baseline (*t* = 0) and follow-up (*t* = 1)
	$\sigma_{j\;g}^{2}$	Variance of infected individuals of study *j* and treatment group *g*
	*v* ^(1)^	Mean egg burden of non-infected individuals
	*r*	Aggregation in non-infected individuals
	*σ*_*d*_	Day-to-day variation in egg counts
	*π*_*jg*_	Prevalence of treatment group *g* of study *j*
	*c*_*jg*_	Cure rate of treatment group *g* of study *j*
	*ϕ*_*jg*_	Egg reduction rate of treatment group *g* of study *j*
	*α*_*jg*_, *β*_*jg*_	Parameters of Gamma distribution describing $\mu_{i_{j\;g}}^{\left(1\right)}$
Worms	*w*_*jg*_	Mean worm burden
	*k*_*w*_	Aggregation of worms
	*z*	number of eggs per fertilized female worm

Let $Y_{i_{jg}ds}^{\left(t\right)}$ be the egg counts (eggs per Kato-Katz slides) for individual *i* from study *j* and treatment group *g*, at measurement day *d* for sample *s* at baseline (*t* = 0) or follow-up (*t* = 1). For the baseline data, we assumed a negative binomial distribution with mean $\mu_{i_{jg}d}^{\left(0\right)}$ and aggregation parameter *k*^(0)^:

$$\begin{array}{c} Y_{i_{jg}ds}^{\left(0\right)}∼\text{NB}\left(\mu_{i_{jg}d}^{\left(0\right)},k^{\left(0\right)}\right). \end{array}$$ (1)

For the follow-up data $Y_{i_{jg}ds}^{\left(1\right)}$ we chose a mixture model to separate the infected from the non-infected individuals. Let $D_{i_{jg}}$ be the disease status of individual *i* (from study *j* and treatment group *g*) defined as

$${\text{D}}_{i_{jg}}=0,\text{if}\;\text{individual}\;i\;\text{harbors}\;\text{no}\;\text{fertilized}\;\text{female}\;\text{worms}$$ (2)

$${\text{D}}_{i_{jg}}=1,\text{if}\;\text{individual}\;i\;\text{harbors}\;\text{at}\;\text{least}\;\text{one}\;\text{fertilized}\;\text{female}\;\text{worm}.$$ (3)

We modeled the likelihood of the egg counts at follow-up with a two component mixture, where the ‘true’ prevalence $\pi_{jg}=\text{P}\left({\text{D}}_{i_{jg}}=1\right)$ denotes the mixture component, that is

$$\text{P}(Y_{i}^{\left(1\right)}|{\text{D}}_{i_{jg}}=0)\equiv \prod_{d,s}NB\left(v^{\left(1\right)},r\right)$$ (4)

$$\text{P}(Y_{i}^{\left(1\right)}|{\text{D}}_{i_{jg}}=1)\equiv \prod_{d,s}NB\left(\mu_{i_{jg}d}^{\left(1\right)},k^{\left(1\right)}\right),$$ (5)

with *v* the mean of the non-infected individuals and *r* the aggregation in the non-infected individuals. We assume conditional independence of the egg counts from the same individual given the disease status [23]. $\mu_{i_{jg}d}^{\left(1\right)}$ is the daily individual mean of the infected individuals and *k*^(1)^ is the variation from slide to slide. We took into account the day-to-day variation ${\left(\sigma_{d}^{\left(t\right)}\right)}^{2}$ in the excreted eggs by day-specific random effects for each *i* and *t*, as follows $log\left(\mu_{i_{jg}d}^{\left(t\right)}\right)=log\left(\mu_{i_{jg}}^{\left(t\right)}\right)+\epsilon_{id}^{\left(t\right)}$ where $\epsilon_{id}^{\left(t\right)}∼N\left(\frac{-{\left(\sigma_{d}^{\left(t\right)}\right)}^{2}}{2},{\left(\sigma_{d}^{\left(t\right)}\right)}^{2}\right).$ The ‘true’ prevalence *π*_*jg*_ was defined as the probability of having at least one fertilized female worm (see Eq 8) and the CR is therefore given by *c*_*jg*_ = 1 − *π*_*jg*_.

For the mean infection intensities, we assumed a Gamma prior distribution. More specifically, at baseline $\mu_{i_{jg}}^{\left(0\right)}∼\text{Gamma}\left(\mu_{jg}^{\left(0\right)}\cdot \sigma_{jg}^{\left(0\right)},\sigma_{jg}^{\left(0\right)}\right)$, where $\mu_{jg}^{\left(0\right)}$ and $\sigma_{jg}^{\left(0\right)}$ are hyperparameters. At follow-up, the mean and the variance of the gamma distribution $\mu_{i_{jg}}^{\left(1\right)}∼\text{Gamma}\left(\alpha_{jg}^{\left(1\right)},\beta_{jg}^{\left(1\right)}\right)$ are obtained from the distribution of the egg counts which is derived by marginalizing the joint distribution of egg counts and fertilized female worms (see S2 Appendix). The joint distribution can be written as the product of the conditional distribution of the egg counts given the feritlized female worms and the distribution of fertilized female worms. The distribution of the fertilized female worms is derived from the distribution of the worms. In particular, we assume that the male and female worms are distributed together [24, 25] and that one male worm can fertilize all female worms. Let *N*_*f*_ be the number of female and *N*_*m*_ the number of male worms, *w*_*jg*_ the mean worm burden in the population and *k*_*w*_ the aggregation of the worms, then the worm distribution is given below:

$$\begin{array}{c} \text{NB(}N_{f},N_{m}\left|w_{jg},k_{w}\right)=\frac{\Gamma (N_{f}+N_{m}+k_{w})}{N_{f}!N_{m}!\Gamma \left(k_{w}\right)}\cdot {\left(1-\alpha \right)}^{k_{w}}\cdot {\left(\alpha p\right)}^{N_{f}}\cdot {\left(\alpha \left(1-p\right)\right)}^{N_{m}}, \end{array}$$ (6)

where $\alpha =\frac{w_{jg}}{w_{jg}+k_{w}}$ and $p=\frac{1}{2}$ is the probability of a worm to be female. It follows that the distribution of fertilized female worms *n*_*f*_ (see S1 Appendix) is

$$\begin{array}{c} P\left(n_{f};w_{jg},k_{w}\right)=\text{NB}(n_{f};\frac{w_{jg}}{2},k_{w})-\text{NB(}n_{f};w_{jg},k_{w})\cdot {\left(\frac{1}{2}\right)}^{n_{f}},\;n_{f}\geq 1. \end{array}$$ (7)

We assume that the conditional distribution of the egg counts given the fertilized female worms is a negative binomial distribution with mean *z* * *n*_*f*_ and aggregation parameter *k*^(1)^, where *z* is a parameter describing the net egg output per fertilized female worm. Due to the low infection intensities the density dependence was not taken into account.

The ‘true’ CR, *c*_*jg*_ is computed from the ‘true’ prevalence that is

$$\begin{array}{c} c_{jg}=P\left(0,w_{jg},k_{w}\right)=2{\left(\frac{k_{w}}{w_{jg}/2+k_{w}}\right)}^{k_{w}}-{\left(\frac{k_{w}}{w_{jg}+k_{w}}\right)}^{k_{w}}. \end{array}$$ (8)

We defined *ϕ*_*jg*_ to be the ERR of treatment *g* and trial *j*. By combining the group-specific infection intensity of the population $\mu_{jg}^{\left(0\right)}$ at baseline, $\mu_{jg}^{\left(1\right)}$ at follow-up, and the prevalence, we were able to compute the ERR *ϕ*_*jg*_ as

$$\begin{array}{c} \phi_{jg}=1-\frac{\mu_{jg}^{\left(1\right)}\cdot \pi_{jg}}{\mu_{jg}^{\left(0\right)}}. \end{array}$$ (9)

Finally, we calculated the posterior distribution of the treatment-specific ERR *ϕ*_*g*_ and CR *c*_*g*_, as a weighted average of the trial specific *ϕ*_*jg*_ and *c*_*jg*_, respectively, with normalized weights proportional to the sample size. For the hyperparameters, the following priors were chosen: for $\mu_{jg}^{\left(0\right)}$ a gamma distribution with mean 50 and variance 1,250; for $\sigma_{jg}^{\left(0\right)}$ an exponential distribution with mean 0.5 and variance 0.25, for ${\left(\sigma_{d}^{\left(t\right)}\right)}^{2}$ a gamma distribution with mean 1 and variance 1; for 1/*k*^(0)^ and 1/*k*^(1)^ normal prior distributions with mean 0 and variance 1; for *w*_*jg*_ a gamma distribution with mean 2 and variance 10; for *k*_*w*_ a normal distribution with mean 0.4 and variance 0.5; for *r* a normal distribution truncated at 0 with mean 0, and variance 1 and for *z* a normal with mean 0.8 and variance 1. If prior knowledge was scarce or ambiguous, weakly informative priors were chosen, otherwise semi-informative priors were applied according to the biological literature [26]. All individuals were included in this analysis as for missing or indeterminate values missing at random (MAR) can be assumed. The model was run in Stan (version 2.19.1) with 10 chains and 10,000 iterations of which the first 5,000 were not included. Convergence was determined with Gelman and Rubin diagnostics [27].

Posterior samples of the day-to-day variation in egg excretion ${\left(\sigma_{d}^{\left(t\right)}\right)}^{2}$ and the egg aggregation *k* were used to obtain the posterior distribution of the sensitivity as a function of infection intensity via the relationship

$$\begin{array}{c} s_{id}=1-\prod_{r=1}^{d}NB{\left(0,\mu_{ir},k\right)}^{s\cdot k}=1-{\left(\frac{k}{\mu_{i1}+k}\right)}^{s\cdot k}\cdot \;\ldots \;\cdot {\left(\frac{k}{\mu_{id}+k}\right)}^{s\cdot k}, \end{array}$$ (10)

where *k* is the posterior mean of the average of *k*^(0)^ and *k*^(1)^. We simulated data for mean infection intensities of 0–500 eggs per gram of stool (EPG) for either one, two, or four Kato-Katz thick smears. For one and two Kato-Katz thick smears it was assumed that the replicate samples were analyzed on the same day, in the case of four Kato-Katz thick smears it was assumed that two samples were analyzed on one day. We considered the above testing regimen as they are the most common ones in clinical trials. Parameter estimates were provided in terms of their posterior median and the 95% Bayesian credible interval (BCI).

## Results

### Descriptive data analysis

Table 1 shows the mean hookworm infection intensities at baseline in the included studies from Côte d’Ivoire, Lao PDR, and Tanzania. All of the infections were classified as light infections (i.e. <2,000 EPG [28]). The sample sizes were similar for the different treatment arms within the individual studies, with exception of the trial in Lao PDR where approximately twice as many children were assigned to two treatment arms (albendazole plus oxantel and albendazole plus oxantel plus pyrantel) [9]. There were slight differences in CRs and ERRs for the same treatments. For instance, there were three trials administering 500 mg mebendazole with CRs of 13%, 18%, and 24% and ERRs of 53%, 45%, and 11%, respectively [20–22]. However for 400 mg tribendimidine, the CRs estimated from three trials were rather similar, i.e. 45%, 55%, and 58%, respectively, whereas the ERRs varied more considerably from 55% to 79%. These results from the descriptive analysis (Table 3) are also reflected in the model estimates as they were informed by the data.

**Table 3:** Estimates of CRs and ERRs among different trials obtained from raw data.

Treatment/dose	CR1	ERR1	CR2	ERR2	CR3	ERR3
Albendazole 400 mg	0.60 (0.51, 0.69)	0.76 (0.54, 0.89)	-	-	-	-
Albendazole 400 mg+ivermectin 200 μg/kg	0.50 (0.35, 0.65)	0.90 (0.69, 0.96)	-	-	-	-
Albendazole 400 mg+mebendazole 500 mg	0.48 (0.34, 0.62)	0.73 (0.27, 0.90)	-	-	-	-
Albendazole 400 mg+oxantel pamoate 20 mg/kg	0.53 (0.45, 0.61)	0.91 (0.86, 0.95)	0.45 (0.33, 0.59)	0.71 (0.41, 0.87)	-	-
Albendazole 400 mg+oxantel pamoate 25 mg/kg	0.37 (0.24, 0.51)	0.82 (0.64, 0.91)	0.54 (0.44, 0.63)	0.91 (0.84, 0.95)	-	-
Albendazole 400 mg+pyrantel pamoate 20 mg/kg+oxantel pamoate 20 mg/kg	0.84 (0.77, 0.89)	0.98 (0.97, 0.99)	-	-	-	-
Albendazole 400 mg+oxantel pamoate 20 mg/kg cons^3^	0.51 (0.42, 0.61)	0.87 (0.68, 0.95)	-	-	-	-
Mebendazole 500 mg	0.18 (0.11, 0.26)	0.45 (0.11, 0.65)	0.24 (0.13, 0.39)	0.11 (-0.76, 0.58)	0.13 (0.07, 0.21)	0.53 (0.29, 0.69)
Mebendazole 500 mg+pyrantel pamoate 20 mg/kg+oxantel pamoate 20 mg/kg	0.70 (0.58, 0.79)	0.93 (0.79, 0.99)	-	-	-	-
Mebendazole 6x100 mg	0.98 (0.93, 1.00)	1.00 (0.99, 1.00)	-	-	-	-
Oxantel pamoate 20 mg/kg	0.11 (0.06, 0.17)	0.14 (-0.39, 0.47)	-	-	-	-
Pyrantel pamoate 20 mg/kg+oxantel pamoate 20 mg/kg	0.51 (0.40, 0.63)	0.96 (0.93, 0.98)	-	-	-	-
Tribendimidine 100 mg	0.21 (0.10, 0.37)	0.27 (-0.64, 0.69)	-	-	-	-
Tribendimidine 200 mg	0.39 (0.23, 0.56)	0.58 (-0.14, 0.83)	-	-	-	-
Tribendimidine 400 mg	0.45 (0.33, 0.59)	0.75 (0.46, 0.89)	0.55 (0.38, 0.71)	0.55 (0.03, 0.82)	0.58 (0.48, 0.68)	0.79 (0.52, 0.93)
Tribendimidine 400 mg+ivermectin 200 μg/kg	0.91 (0.81, 0.96)	0.94 (0.86, 0.99)	0.81 (0.72, 0.88)	0.98 (0.95, 0.99)	-	-
Tribendimidine 400 mg+oxantel pamoate 25 mg/kg	0.51 (0.38, 0.64)	0.76 (0.54, 0.90)	0.53 (0.43, 0.62)	0.85 (0.77, 0.91)	-	-

^1^ CR = cure rate; CR1, CR2, and CR3 refer to data from individual studies

^2^ ERR = egg reduction rate; ERR1, ERR2, and ERR3 refer to data from individual studies

^3^ Administered on 2 consecutive days

### Model-based estimates

The estimated CRs ranged from 10% to 88% and the ERRs from −26% to 100% (Table 4).

**Table 4:** Posterior estimates (mean, 95% BCI) for CR and ERR.

Treatment	Cure rate (*c*_*g*_)	Egg reduction rate (*ϕ*_*g*_)
Albendazole 400 mg	0.44 (0.37,0.51)	0.74 (0.57,0.86)
Albendazole 400 mg+ivermectin 200 μg/kg	0.41 (0.31,0.52)	0.79 (0.55,0.91)
Albendazole 400 mg+mebendazole 500 mg	0.35 (0.25,0.45)	0.68 (0.35,0.86)
Albendazole 400 mg+oxantel pamoate 20 mg/kg	0.38 (0.32,0.43)	0.83 (0.74,0.90)
Albendazole 400 mg+oxantel pamoate 20 mg/kg^1^	0.38 (0.31,0.45)	0.77 (0.63,0.87)
Albendazole 400 mg+oxantel pamoate 25 mg/kg	0.35 (0.30,0.41)	0.82 (0.72,0.88)
Albendazole 400 mg+pyrantel pamoate 20 mg/kg+oxantel pamoate 20 mg/kg	0.66 (0.59,0.73)	0.98 (0.97,0.99)
Mebendazole 500 mg	0.14 (0.11,0.18)	0.21 (-0.05,0.41)
Mebendazole 500 mg+pyrantel pamoate 20 mg/kg+oxantel pamoate 20 mg/kg	0.52 (0.43,0.61)	0.96 (0.91,0.98)
Mebendazole 6x100 mg	0.88 (0.79,0.95)	1.00 (0.98,1.00)
Oxantel pamoate 20 mg/kg	0.10 (0.07,0.15)	-0.26 (-0.80,0.16)
Pyrantel pamoate 20 mg/kg+oxantel pamoate 20 mg/kg	0.39 (0.3,0.47)	0.94 (0.9,0.97)
Tribendimidine 100 mg	0.18 (0.1,0.28)	0.13 (-0.71,0.62)
Tribendimidine 200 mg	0.27 (0.17,0.39)	0.26 (-0.56,0.71)
Tribendimidine 400 mg	0.39 (0.34,0.45)	0.72 (0.58,0.83)
Tribendimidine 400 mg+ivermectin 200 μg/kg	0.67 (0.60,0.73)	0.95 (0.89,0.98)
Tribendimidine 400 mg+oxantel pamoate 25 mg/kg	0.38 (0.32,0.44)	0.79 (0.67,0.87)

^1^ Administered on 2 consecutive days

The treatments that achieved the highest ‘true’ CRs and ERRs against hookworm infections were mebendazole (6x100 mg) with a CR of 88% (95% BCI 79–95%) and an ERR of 99.6% (95% BCI 98–100%) and the triple combination treatment (albendazole 400 mg plus oxantel pamoate 20 mg/kg plus pyrantel pamoate 20 mg/kg) with a CR of 66% (95% BCI 59–73%) and an ERR of 98% (95% BCI 97–99%). Tribendimidine (400 mg) plus ivermectin (200 μg/kg) resulted in a CR of 67% (95% BCI 60–73%) and an ERR of 95% (95% BCI 89–98%).

**Fig 2:**
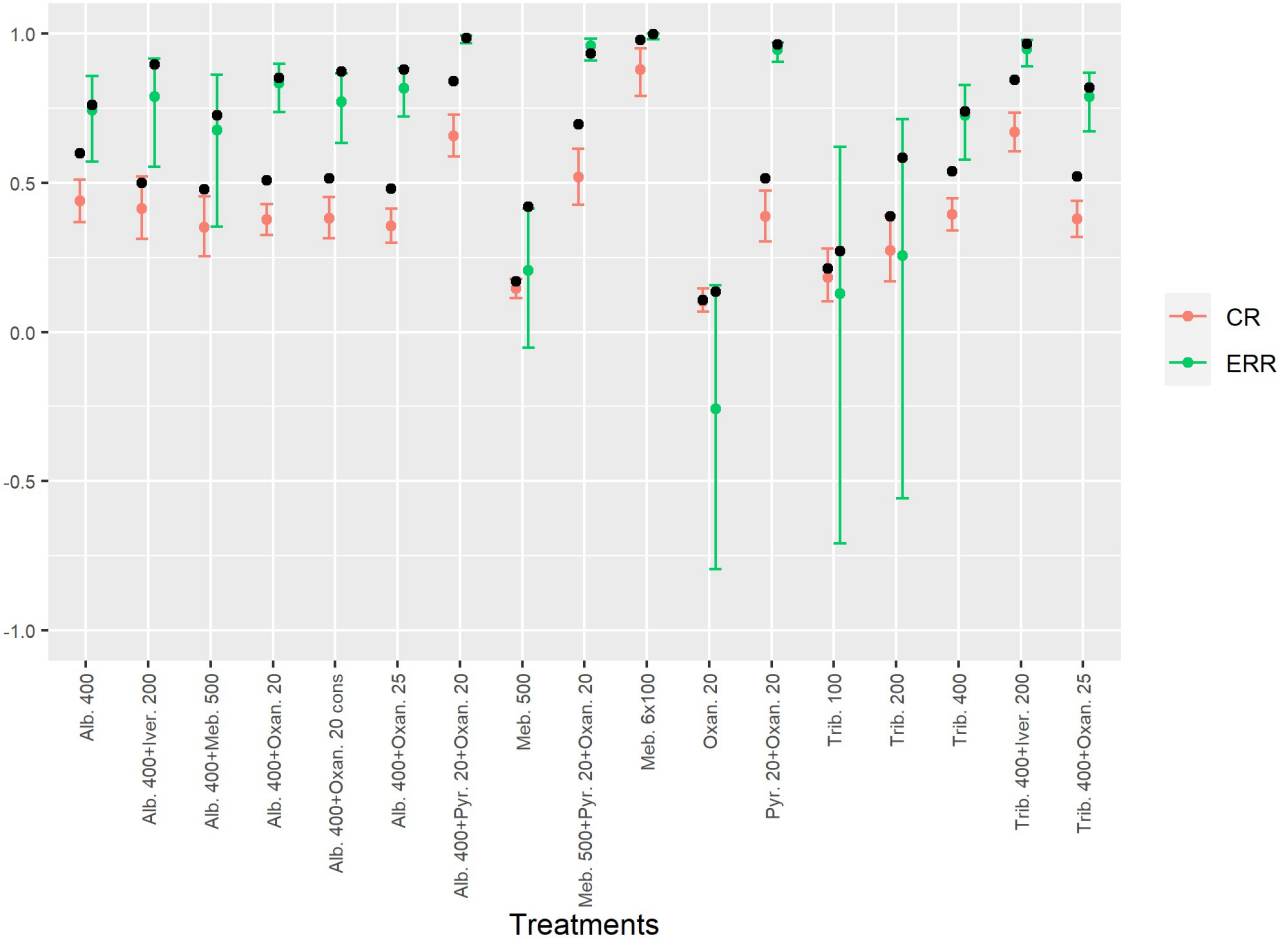
Estimated and observed cure rates (CRs) and egg reduction rates (ERRs). Posterior mean and 95% Bayesian credible interval of the CR and the ERR (arithmetic mean) for the different treatment arms for hookworm. The black dots show the observed data.

The two least efficacious treatments were mebendazole 500 mg and oxantel pamoate (20 mg/kg) with CRs of 14% (95% BCI 11–18%) and 10% (95% BCI 7–15%) and ERRs of 21% (95% BCI -5–41%) and -26% (95% BCI -8–16%), respectively. Fig 2 shows a comparison of the model estimates of the CRs and ERRs to the observed ones, where the ‘true’ estimates of CR were lower than the observed ones. However, ‘true’ estimates of ERR were similar to the observed ones. The model estimates of the CR, ERR, variation at baseline and aggregation at follow-up for every treatment arm and trial and the mean intensities at baseline and follow-up are provided in S1 Table and S1 Fig, respectively. The estimate of the egg output per fertilized female worm was 1.97 (95% BCI 1.58–2.43).

### Diagnostic sensitivity

**Fig 3:**
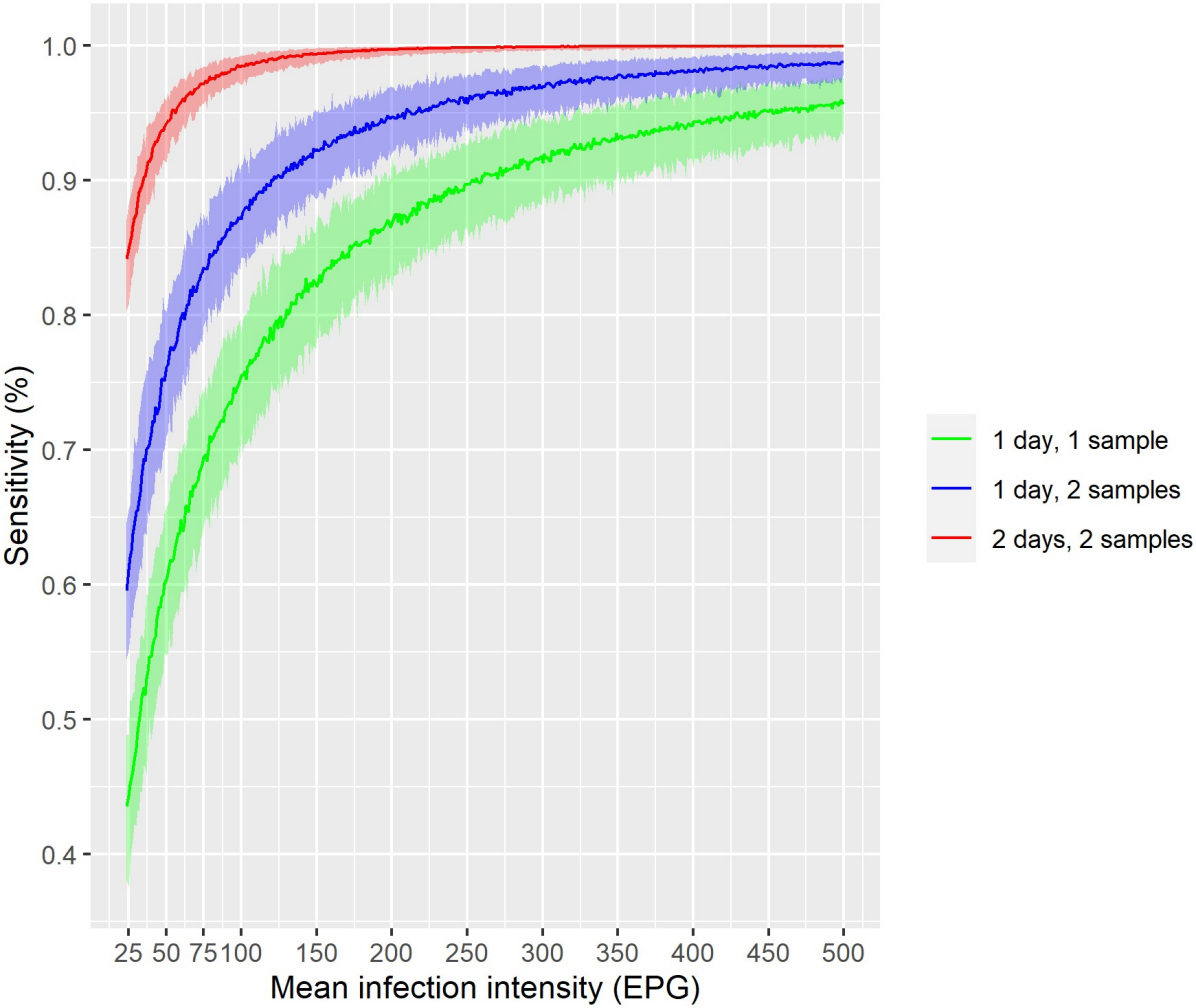
Sensitivity of the Kato-Katz thick smear technique for hookworm for one, two, and four Kato-Katz thick smears. The lines show the posterior mean estimate and the shaded areas indicate the 95% Bayesian credible interval.

The estimate of the day-to-day variation in egg excretion ${\left(\sigma_{d}^{\left(t\right)}\right)}^{2}$ was 1.19 (95% BCI 1.15–1.25) and of the egg aggregation parameter at baseline *k*^(0)^ was 9.68 (95% BCI 8.79–10.63) and at follow-up *k*^(1)^ 11.99 (95% BCI 10.03–14.1). The posterior distribution of the difference has mean 1.95 (95% BCI 0.13–3.63) indicating that the aggregation parameters differ at baseline and follow-up as 0 is not included in BCI. Estimates of the sensitivity of the Kato-Katz thick smear technique are shown in Fig 3. For ‘true’ intensities of an individual with a hookworm infection above 50 EPG, the sensitivity was above 92% for four Kato-Katz thick smears obtained from two stool specimens. For two Kato-Katz thick smears the sensitivity dropped to between 72% and 80% and in the case of only a single Kato-Katz thick smear it ranged between 55% and 65%. For hookworm infection intensities of more than 350 EPG, the sensitivity was above 90% irrespective of the number of Kato-Katz thick smears examined. As the estimated sensitivity from the study of Bärenbold et al. (2017) [16] was different to ours, we implemented that model and ran it with the data used in this analysis. We obtained similar results for the sensitivity as in the prior work by Bärenbold et al. (2017) [16] (see S2 Fig).

## Discussion

This is the first model-based meta-analysis of the effects of different drugs and treatment regimen against hookworm infection, which takes into account the diagnostic error of the widely used Kato-Katz thick smear technique. Using Bayesian inferences, we estimated the CR and ERR of 17 different treatments, the infection intensity dependent sensitivity, and the day-to-day variation of the excreted hookworm eggs. All trials included in the meta-analysis were conducted with the same diagnostic procedures and the transmission model was fitted on individual-level egg count data. Our work is in contrast to other analyses based on aggregated data, which suffer from a lack of comparability because of different diagnostic procedures or different summary measures of treatment group intensity. Due to low diagnostic sensitivity of the Kato-Katz technique, it is more likely to miss infections of individuals with low intensities, and hence, overestimate CRs [29]. Our analysis addresses this issue, and hence, our ‘true’ CR estimates are lower than the observed CRs (Fig 2). This is reflected not only in the point estimates, but also in the 95% BCIs of the model-based CRs, which are shifted compared to the confidence intervals of the CRs computed from the raw data. This indicates that the bias due to diagnostic error is quite large. Nevertheless, it is in line with other studies, where Kato-Katz results are compared to polymerase chain reaction (qPCR) results. For instance, Keller et al. (2020) reported CRs of the combination treatment with ivermectin plus albendazole based on Kato-Katz of 78.3% compared to 52.4% for qPCR [30]. Barda et al. (2020) reported similar CR differences for albendazole, namely 77.8% for Kato-Katz vs 57.1% for qPCR [31]. In the study of Benjamin-Chung et al. (2020) the participants were also treated with a single dose of albendazole and they also observed much higher CRs when derived from Kato-Katz compared to qPCR of 92.5% vs 78.6%, respectively [32].

Our results confirm earlier findings that treatments which consist of a combination of drugs or multiple doses are more efficacious than a single-drug treatment [11]. Mebendazole administered over 3 days in six dosages of 100 mg each is the most efficacious treatment in terms of both CR and ERR (Table 4). Eshetu et al. (2020) reported similar CRs and ERRs [33], as modeled data; however, participants in those included trials were chracterized by slightly higher infection intensities at baseline (1,134 EPG compared to 465 EPG in our case), which were less affected by diagnostic error. The value of 1 for the ERR of aformentioned treatment arm in Table 4 (corresponding to 100%) is a rounded value and therefore reliable. Moreover, ERRs close to 100% and low CRs can be observed in the field, as in the data for the combination treatment pyrantel pamoate and oxantel pamoate analyzed here [9]. This is the case when many patients have very low egg counts after treatment.

The combination of albendazole, pyrantel pamoate, and oxantel pamoate shows also a very high efficacy in our analysis, as expected. Yet, we obtained a lower CR than reported in a previous meta-analysis [11]. Tribendimine plus ivermectin showed a slightly lower ‘true’ CR and a lower, but still high ERR compared to the aforementioned albendazole combination treatment.

On the other hand, our estimates confirm the low efficacy of the WHO recommended drug mebendazole against hookworm infection when used as a single dose. We estimated CRs and ERRs that are similar or lower to those reported in the literature [11].

Moser et al. (2018) carried out a Bayesian analysis of a subset of the data of the trials conducted in Tanzania and Côte d’Ivoire (see Table 1 trial Moser et al. 2016 [9]), as they only included those individuals who were tested with FECPAK^G2^ [17]. Their estimates for the ERR are similar to ours, however our estimates for the CR are lower, which could be due to improvements in the assumptions of our model.

Our results show that the diagnostic sensitivity increases with the sampling effort. Nevertheless, WHO recommends to collect only one stool sample to be subjected to a single Kato-Katz thick smear, which can have implications, as the CR and ERR are underestimated. Comparing our results to the estimates of Bärenbold et al. (2017) [16], we found a higher sensitivity across all levels of infection intensity, indicating that the sensitivity varies among studies because of different day-to-day variation. This in turn can have various reasons, like accuracy of the readings or variation in egg density of different samples from an individual. For example, for 200 EPG, the sensitivity is close to 87% for one Kato-Katz thick smear and 94% for two Kato-Katz thick smears, whereas in aforementioned analysis the estimates are only roughly 51% and 75%, respectively. This finding was not expected and suggests that treatment efficacy estimates may not be comparable even from studies with similar infection intensities and sampling efforts. A first step to clarify this could be to investigate whether the day-to-day variation depends on the mean infection intensity.

Our study has several limitations. First, we addressed uncertainty by linking the mean infection intensity at follow-up with the aggregation of the worms to improve model fitting. The aggregation parameter of the worms in the population is estimated well but with considerable uncertainty, although we linked the aggregation parameter to the mean infection intensity and the prevalence [34]. Furthermore, there were treatment arms where the estimates of the ERR have a rather large uncertainty compared to the estimates of the mean infection intensity at baseline and follow-up. This is the case for low ERRs. Due to the limited number of trials per treatment, we were unable to include a random effect to account for the variation between the trials [35]. Moreover, we didn’t take into account the density-dependent fecundity of female worms, nevertheless, in this framework this assumption is justified, as only individuals with light infections are analyzed. For settings with higher intensities it should be included.

### Conclusion

We developed a Bayesian model including the distribution of the worms which enabled us to directly compare the treatment effect of different drugs and treatment regimen against hookworm taking into account diagnostic error. We also estimated the diagnostic sensitivity of the Kato-Katz thick smear technique. Despite considerably lower CRs obtained by our modeling framework, our results confirm earlier findings that treatments which consist of a combination of drugs or multiple doses are more efficacious than a single-drug treatment. Moreover, we found that diagnostic sensitivity increases considerably if two stool samples are collected on consecutive days instead of only one or if multiple Kato-Katz thick smears are prepared from a single stool sample and examined under a microscope. Hence, we recommend to collect two stool samples on consecutive days. Furthermore, the comparison of our results to a similar work indicates that the diagnostic sensitivity of the Kato-Katz thick smear technique can vary considerably across studies. The modeling framework used here could be adapted for the other helminth species parasitizing humans and animals.

## Supporting information

S1 Appendix Derivation of the distribution of fertilized female worms and the corresponding prevalence. (PDF)

S2 Appendix Mean and variance of the distribution of fertilized female worms. (DOCX)

S3 Appendix Posterior distribution. (PDF)

S1 Table Parameter estimates for each treatment regimen of all trials for hookworm. (PDF)

S1 Fig The plot shows the estimated mean and 95% Bayesian credible interval for the mean egg intensity at baseline and follow-up for the different treatment arms for hookworm. The black dots show the data.
(TIFF)

S2 Fig Sensitivity of the Kato-Katz technique for hookworm for one, two, and three samples. The lines show the mean sensitivity and the shaded areas indicate the 95% BCI.
(TIFF)

S1 STARD Checklist (DOCX)
