# Supplementary material for: Modeling the effect of different drugs and treatment regimen for hookworm on cure and egg reduction rates taking into account diagnostic error
Source: PLoS Negl Trop Dis. 2022 Oct 4;16(10):e0010810. doi: 10.1371/journal.pntd.0010810 (PMC9595538; doi:10.1371/journal.pntd.0010810)
Supplement: S1 Appendix — (PDF) [file pntd.0010810.s001.pdf]

S1 Appendix: Derivation of the marginal distribution of mated female worms

The probability of having N_f female and N_m male worms is according to May and Woolhouse [1] defined as follows

$$P(N_f, N_m; q, p, w_{jg}, k_w) = (1-\alpha)^{k_w} \frac{\Gamma(N_f + N_m + k_w)}{\Gamma(k_w)} \frac{(\alpha q)^{N_f}}{N_f!} \frac{(\alpha p)^{N_m}}{N_m!}, \quad \alpha = \frac{w_{jg}}{w_{jg} + k_w}$$

where q is the probablity of a worm to be female, p to be male, w_{jg} is the mean worm burden and k_w the aggregation parameter of a negative binomial distribution of the worms in the population. We assume that the probability for a worm to be female is $q = 1/2$ and $p = 1 - q = 1/2$ to be male. Moreover, we assume that one male worm can fertilize all female worms.

To derive the marginal distribution of the female worms we sum over the number of male worms

$$\begin{aligned} P(N_f; q, w_{jg}, k_w) &= \frac{(1-\alpha)^{k_w}}{\Gamma(k_w)} \frac{(\frac{\alpha}{2})^{N_f}}{N_f!} \sum_{N_m=1}^{\infty} \frac{\Gamma(N_f + N_m + k_w) \cdot (\frac{\alpha}{2})^{N_m}}{N_m!} \\ &= -\frac{(1-\alpha)^{k_w}}{\Gamma(k_w)} \frac{(\frac{\alpha}{2})^{N_f}}{N_f!} \frac{\Gamma(k_w + N_f + 1) \cdot (1 - \frac{\alpha}{2})^{-k_w - N_f} \cdot (-1 + (1 - \frac{\alpha}{2})^{-k_w - N_f})}{k_w + N_f} \\ &= \frac{(1-\alpha)^{k_w}}{\Gamma(k_w)} \frac{(\frac{\alpha}{2})^{N_f}}{N_f!} \Gamma(k_w + N_f)(1 - \frac{\alpha}{2})^{-k_w - N_f} - \Gamma(k_w + N_f). \end{aligned} \quad (1)$$

Now we use the relations $(1 - \alpha) = \frac{k_w}{w_{jg} + k_w}$ and $\left(1 - \frac{\alpha}{2}\right)^{-1} = \frac{2(w_{jg} + k_w)}{w_{jg} + 2k_w}$ in equation 1:

$$\frac{(1-\alpha)^{k_w}(\frac{\alpha}{2})^{N_f}}{\Gamma(k_w)N_f!}\cdot\left[\Gamma(k_w+N_f)\left(1-\frac{\alpha}{2}\right)^{-k_w-N_f}-\Gamma(k_w+N_f)\right]$$
$$=\frac{\Gamma(k_w+N_f)}{\Gamma(k_w)N_f!}\left(\frac{k_w}{w_{jg}+k_w}\frac{2(w_{jg}+k_w)}{w_{jg}+2k_w}\right)^{k_w}\left(\frac{w_{jg}}{2(w_{jg}+k_w)}\frac{2(w_{jg}+k_w)}{w_{jg}+2k_w}\right)^{N_f}$$
$$-\frac{\Gamma(k_w+N_f)}{\Gamma(k_w)N_f!}\left(\frac{k_w}{w_{jg}+k_w}\right)^{k_w}\left(\frac{w_{jg}}{2(w_{jg}+k_w)}\right)^{N_f}$$
$$=\frac{\Gamma(k_w+N_f)}{\Gamma(k_w)N_f!}\left(\frac{2k_w}{w_{jg}+2k_w}\right)^{k_w}\left(\frac{w_{jg}}{w_{jg}+2k_w}\right)^{N_f}-\frac{\Gamma(k_w+N_f)}{\Gamma(k_w)N_f!}\left(\frac{k_w}{w_{jg}+k_w}\right)^{k_w}\left(\frac{w_{jg}}{2(w_{jg}+k_w)}\right)^{N_f}$$
$$=\frac{\Gamma(k_w+N_f)}{\Gamma(k_w)N_f!}\left(\frac{k_w}{w_{jg}/2+k_w}\right)^{k_w}\left(\frac{w_{jg}/2}{w_{jg}/2+k_w}\right)^{N_f}$$
$$-\frac{\Gamma(k_w+N_f)}{\Gamma(k_w)N_f!}\left(\frac{k_w}{w_{jg}+k_w}\right)^{k_w}\left(\frac{w_{jg}}{w_{jg}+k_w}\right)^{N_f}\left(\frac{1}{2}\right)^{N_f}$$
$$=\mathrm{NB}(N_f;w_{jg}/2,k_w)-\mathrm{NB}(N_f;w_{jg},k_w)\left(\frac{1}{2}\right)^{N_f}$$
$$=\mathrm{P}(N_f;w_{jg},k_w).$$

We consider that all female worms are fertilized when $N_m > 0$ and therefore we replace N_f by n_f.

To compute the prevalence of having at least one fertilized female worm, we need to compute the probability of having no male worms, no female worms and no worms of either sex, which are given in equations 2, 3 and 4, respectively

$$\sum_{N_m=0}^{\infty} P(N_f,0;q=1/2,w_{jg},k_w)=\left(\frac{k_w}{w_{jg}/2+k_w}\right)^{k_w} \tag{2}$$

$$\sum_{N_f=0}^{\infty} P(0,N_m;p=1/2,w_{jg},k_w)=\left(\frac{k_w}{w_{jg}/2+k_w}\right)^{k_w} \tag{3}$$

$$P(0,0;q=1/2,p=1/2,w_{jg},k_w)=\left(\frac{k_w}{w_{jg}+k_w}\right)^{k_w}. \tag{4}$$

As equations 2 and 3 already contain the probabilty of having no worms, the last term of equation 4 accounts for it in the following definition of the prevalence

$$1-P(0;w_{jg},k_w)=1-2\left(\frac{k_w}{w_{jg}/2+k_w}\right)^{k_w}+\left(\frac{k_w}{w_{jg}+k_w}\right)^{k_w}.$$

References

[1] May, R. M., & Woolhouse, M. E. J. Biased sex ratios and parasite mating probabilities. Parasitology. 1993; 107(3), 287-295.
