# Supplementary material for: Modeling the effect of different drugs and treatment regimen for hookworm on cure and egg reduction rates taking into account diagnostic error
Source: PLoS Negl Trop Dis. 2022 Oct 4;16(10):e0010810. doi: 10.1371/journal.pntd.0010810 (PMC9595538; doi:10.1371/journal.pntd.0010810)
Supplement: S2 Appendix — (DOCX) [file pntd.0010810.s002.docx]

**S2 Appendix: Mean and variance of the distribution of egg counts**

The marginal distribution of egg counts (i.e. $\boldsymbol{Y}_{i}^{\left( 1 \right)}$at follow-up) can be computed from the joint distribution of egg counts and fertilized female worms $n_{f}$as follows

$P\left( \boldsymbol{Y}_{i}^{\left( 1 \right)} \right)=\sum_{n_{f}=0}^{\infty} P(\boldsymbol{Y}_{i}^{\left( 1 \right)},n_{f})=\sum_{n_{f}=0}^{\infty} P\left( \boldsymbol{Y}_{i}^{\left( 1 \right)} | n_{f} \right)P(n_{f;}w_{jg},k_{w})$ (S2.1)

The conditional distribution is $P\left( \boldsymbol{Y}_{i}^{\left( 1 \right)} | n_{f} \right)\equiv NB\left( zn_{f},k^{(1)} \right)$, where z is the net egg output of fertilized female worms and $k^{(1)}$ is the aggregation of the egg counts at follow-up. $P(n_{f;}w_{jg},k_{w})$ is the distribution of fertilized female worms, where $w_{jg}$ is the mean worm burden and $k_{w}$ is the aggregation of the worms in the population. The mean and variance of the distribution of the egg counts in (1) are

$$\mu_{jg}^{(1)}=\sum_{n_{f}=0}^{\infty} z n_{f} P(n_{f};w_{jg},k_{w})=\sum_{n_{f}=0}^{\infty} z n_{f}\left( NB(n_{f};w_{jg}/2,k_{w})-NB(n_{f};w_{jg},k_{w})\left( \frac{1}{2} \right)^{n_{f}} \right)=\frac{w_{jg} z}{2}-2^{k_{w}} w_{jg} \left( \frac{k_{w}}{2 k_{w}+w_{jg}} \right)^{1+k_{w}} z$$

$$\sigma_{jg}^{2}=\left( 1+\frac{1}{k^{(1)}} \right)\left( \sum_{n_{f}=0}^{\infty} z^{2} {n_{f}}^{2}\left( NB\left( n_{f};\frac{w_{jg}}{2},k_{w} \right)-NB\left( n_{f};w_{jg},k_{w} \right)\left( \frac{1}{2} \right)^{n_{f}} \right) \right)-\left( \mu_{jg}^{\left( 1 \right)} \right)^{2}+\mu_{jg}^{(1)}$$

$$=\left( 1+\frac{1}{k^{(1)}} \right)\left( \frac{\left( \frac{w_{jg}}{2} \right)\left( 2k+w_{jg}+k_{w}w_{jg} \right)z^{2}}{2k_{w}}-\frac{k_{w}w_{jg}\left( \frac{k_{w}}{k_{w}+w_{jg}} \right)^{k_{w}}\left( 2k_{w}+2w_{jg}+k_{w}w_{jg} \right)\left( 1-\frac{w_{jg}}{2\left( k_{w}+w_{jg} \right)} \right)^{-k_{w}}z^{2}}{\left( 2k_{w}+w_{jg} \right)^{2}} \right)-\left( \mu_{jg}^{\left( 1 \right)} \right)^{2}+\mu_{jg}^{(1)}$$
