# Supplementary material for: Modeling the effect of different drugs and treatment regimen for hookworm on cure and egg reduction rates taking into account diagnostic error
Source: PLoS Negl Trop Dis. 2022 Oct 4;16(10):e0010810. doi: 10.1371/journal.pntd.0010810 (PMC9595538; doi:10.1371/journal.pntd.0010810)
Supplement: S3 Appendix — (PDF) [file pntd.0010810.s003.pdf]

S3 Appendix: Posterior distribution

In the following the posterior distribution

$$P(\text{model parameters}|\text{data}) \propto P(\text{data}|\text{model parameters}) \cdot P(\text{model parameters})$$
$$\Longleftrightarrow P(\boldsymbol{\theta}|D) \propto P(D|\boldsymbol{\theta}) \cdot P(\boldsymbol{\theta})$$

of the model used in this analysis is presented (see table 2 in the model section of the paper for the definition of the parameters):

$$P(\mu_{jg}^{(0)}, \sigma_{jg}^{(0)}, k^{(0)}, \sigma_{d}^{(0)}, w_{jg}, k_w, k^{(1)}, v^{(1)}, r, \pi_{jg}, \sigma_{d}^{(1)} | \boldsymbol{Y}^{(0)}, \boldsymbol{Y}^{(1)})$$
$$\propto \prod_i P(Y_i{}^{(0)}|\mu_{i_{jg}d}^{(0)}, \mu_{i_{jg}}^{(0)}, \mu_{jg}^{(0)}, \sigma_{jg}^{(0)}, \epsilon_{id}^{(0)}, k^{(0)}) P(\epsilon_{id}^{(0)}|\sigma_{d}^{(0)})$$
$$P(Y_i{}^{(1)}|\mu_{i_{jg}d}^{(1)}, \mu_{i_{jg}}^{(1)}, \mu_{jg}^{(1)}, \sigma_{jg}^{(1)}, w_{jg}, k_w, \epsilon_{id}^{(1)}, k^{(1)}, v^{(1)}, r, \pi_{jg}) P(\epsilon_{id}^{(1)}|\sigma_{d}^{(1)})$$
$$P(\mu_{jg}^{(0)}) P(\sigma_{jg}^{(0)}) P(k^{(0)}) P(\sigma_{d}^{(0)}) P(w_{jg}) P(k_w) P(k^{(1)}) P(v^{(1)}) P(r) P(\pi_{jg}) P(\sigma_{d}^{(1)})$$
