# Supplementary material for: Modeling the effect of different drugs and treatment regimen for hookworm on cure and egg reduction rates taking into account diagnostic error
Source: PLoS Negl Trop Dis. 2022 Oct 4;16(10):e0010810. doi: 10.1371/journal.pntd.0010810 (PMC9595538; doi:10.1371/journal.pntd.0010810)
Supplement: S1 Table — (PDF) [file pntd.0010810.s004.pdf]

S1 Table. Parameter estimates for each treatment regimen of all trials for Hookworm

Treatment	Cure rate (c_g)	Egg reduction rate (ϕ_g)	Aggregation in group at BL ($\sigma_g^{(0)}$)	Aggregation in group at FU (k_w)
Alb 400+Oxan 25[1]	0.26 (0.18,0.35)	0.75 (0.55,0.88)	0.04 (0.02,0.08)	0.99 (0.58,1.58)
Alb 400+Oxan 25b[6]	0.4 (0.33,0.48)	0.85 (0.74,0.91)	0.07 (0.03,0.12)	1.05 (0.58,1.69)
Alb. 400+Oxan. 20b[5]	0.36 (0.28,0.46)	0.63 (0.31,0.82)	0.15 (0.05,0.35)	1.06 (0.57,1.72)
Alb. 400[4]	0.44 (0.37,0.51)	0.74 (0.57,0.86)	0.16 (0.08,0.31)	0.89 (0.48,1.5)
Alb. 400+Meb. 500[5]	0.35 (0.25,0.45)	0.68 (0.35,0.86)	0.1 (0.03,0.25)	0.88 (0.46,1.5)
Alb. 400+Oxan. 20[3]	0.38 (0.32,0.44)	0.92 (0.86,0.95)	0.08 (0.03,0.29)	0.6 (0.39,0.95)
Alb. 400+Oxan. 20[4]	0.38 (0.31,0.45)	0.77 (0.63,0.87)	0.11 (0.06,0.21)	1.09 (0.63,1.74)
Alb. 400mg+Iver 200[5]	0.41 (0.31,0.52)	0.79 (0.55,0.91)	0.08 (0.03,0.2)	1.03 (0.51,1.74)
Alb.400+Pyr.20+Oxan.20[3]	0.66 (0.59,0.73)	0.98 (0.97,0.99)	0.05 (0.02,0.1)	0.49 (0.18,1.15)
Meb 500c[8]	0.11 (0.07,0.16)	0.41 (0.13,0.62)	0.08 (0.04,0.15)	1.57 (1.11,2.14)
Meb 6x100[8]	0.88 (0.79,0.95)	1 (0.98,1)	0.12 (0.05,0.3)	0.53 (0.04,1.38)
Meb. 500[4]	0.15 (0.11,0.2)	0.18 (-0.23,0.48)	0.08 (0.04,0.14)	1.4 (0.97,1.94)
Meb. 500b[5]	0.21 (0.13,0.3)	-0.17 (-1.14,0.44)	0.25 (0.07,0.73)	1.19 (0.72,1.81)
Meb.500+Pyr.20+Oxan.20[3]	0.52 (0.43,0.61)	0.96 (0.91,0.98)	0.13 (0.02,0.71)	0.54 (0.24,1.13)
Oxan. 20[4]	0.1 (0.07,0.15)	-0.26 (-0.8,0.16)	0.18 (0.09,0.37)	1.61 (1.16,2.16)
Pyr. 20+Oxan. 20[3]	0.39 (0.3,0.47)	0.94 (0.9,0.97)	0.13 (0.02,0.74)	1.03 (0.56,1.7)
Trib 100[2]	0.18 (0.1,0.28)	0.13 (-0.71,0.62)	0.13 (0.03,0.47)	0.94 (0.56,1.47)
Trib 200[2]	0.27 (0.17,0.39)	0.26 (-0.56,0.71)	0.19 (0.04,0.65)	0.85 (0.46,1.43)
Trib 400[1]	0.34 (0.25,0.44)	0.7 (0.42,0.86)	0.07 (0.03,0.17)	0.75 (0.41,1.28)
Trib 400+Iver 200[1]	0.73 (0.62,0.84)	0.93 (0.78,0.99)	0.08 (0.03,0.18)	0.2 (0.06,0.65)
Trib 400+Iver 200b[6]	0.64 (0.56,0.71)	0.96 (0.92,0.98)	0.08 (0.04,0.15)	0.82 (0.31,1.56)
Trib 400+Oxan 25[1]	0.36 (0.27,0.46)	0.75 (0.51,0.89)	0.1 (0.03,0.29)	0.65 (0.36,1.12)
Trib 400+Oxan 25b[6]	0.39 (0.31,0.46)	0.81 (0.67,0.89)	0.08 (0.04,0.16)	0.9 (0.5,1.5)
Trib 400b[6]	0.42 (0.35,0.5)	0.81 (0.65,0.9)	0.09 (0.04,0.18)	0.67 (0.37,1.19)
Trib 400c[2]	0.39 (0.27,0.52)	0.5 (-0.11,0.82)	0.4 (0.07,1.43)	0.58 (0.28,1.09)

[1] CIV (2016)
[2] CIV (2017)
[3] LAO (2017)
[4] TAN (2012)
[5] TAN (2013)
[6] TAN (2016)
[7] TAN (2017a)
