# Supplementary figures and images for: Modeling the effect of different drugs and treatment regimen for hookworm on cure and egg reduction rates taking into account diagnostic error
Source: PLoS Negl Trop Dis. 2022 Oct 4;16(10):e0010810. doi: 10.1371/journal.pntd.0010810 (PMC9595538; doi:10.1371/journal.pntd.0010810)

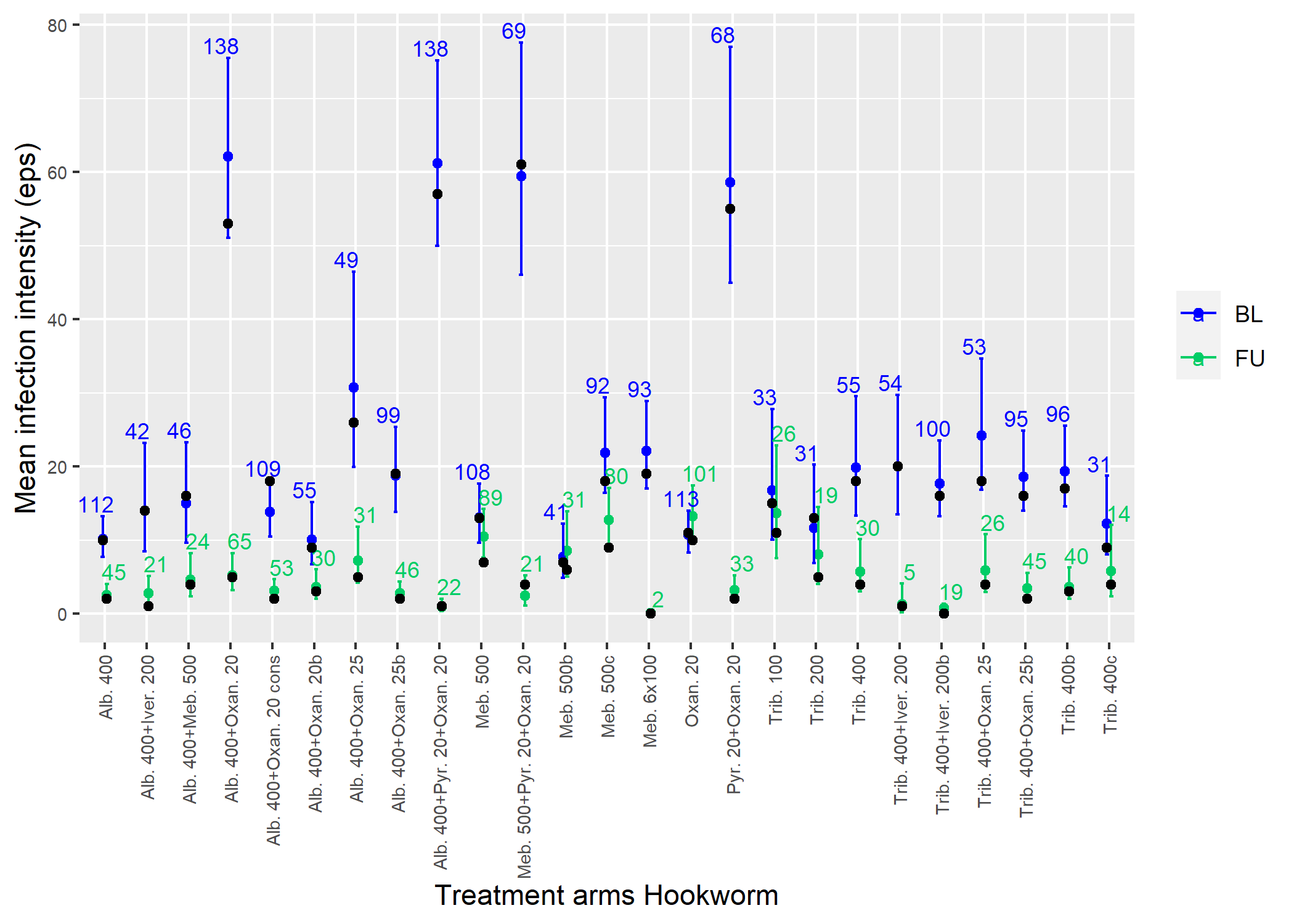

Supplement: S1 Fig — The black dots show the data. (TIFF) [file pntd.0010810.s005.tiff]

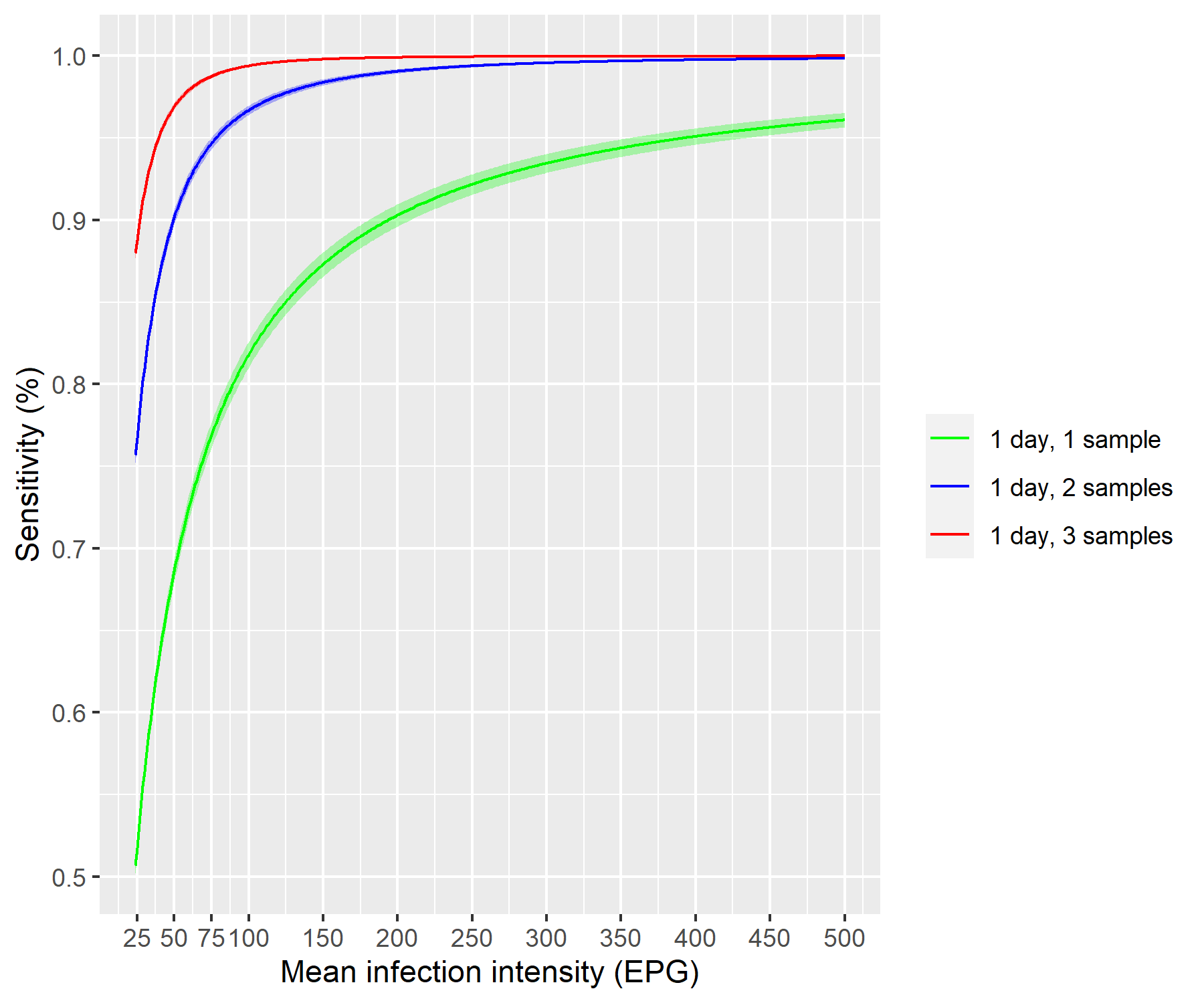

Supplement: S2 Fig — The lines show the mean sensitivity and the shaded areas indicate the 95% BCI. (TIFF) [file pntd.0010810.s006.tiff]
