# Supplementary material for: Modeling the effect of different drugs and treatment regimen for hookworm on cure and egg reduction rates taking into account diagnostic error
Source: PLoS Negl Trop Dis. 2022 Oct 4;16(10):e0010810. doi: 10.1371/journal.pntd.0010810 (PMC9595538; doi:10.1371/journal.pntd.0010810)
Supplement: S1 STARD Checklist — (DOCX) [file pntd.0010810.s007.docx]

|  | **Section & Topic** | **No** | **Item** | **Reported on page #** |
| --- | --- | --- | --- | --- |
|  |  |  |  |  |
|  | **TITLE OR ABSTRACT** |  |  |  |
|  |  | **1** | Identification as a study of diagnostic accuracy using at least one measure of accuracy  (such as sensitivity, specificity, predictive values, or AUC)  **Text excerpts**: -Modeling the effect of different drugs and treatment regimen  for hookworm on cure and egg reduction rates taking into account diagnostic error  -Furthermore, the model accounted for infection intensity dependent sensitivity and day-to-day variation of hookworm egg excretion. | p. 1,2 |
|  | **ABSTRACT** |  |  |  |
|  |  | **2** | Structured summary of study design, methods, results, and conclusions  (for specific guidance, see STARD for Abstracts)  Methodology, Principal findings and Conclusions from Abstract | p. 1,2 |
|  | **INTRODUCTION** |  |  |  |
|  |  | **3** | Scientific and clinical background, including the intended use and clinical role of the index test  **Text excerpts**: Systematic reviews and meta-analyses have been carried out to compare the efficacy of different treatments against hookworm and other STH infections [1-3]. However, most trials assess the presence of hookworms using the Kato-Katz thick smear technique, which has low sensitivity, and hence, the efficacy of the treatment is overestimated.  - The model has been successfully applied to data from a clinical trial on Pemba island to assess the performance of FECPAKG2 and the Kato-Katz thick smear technique for the diagnosis of STH infection [4]. | p. 4,5 |
|  |  | **4** | Study objectives and hypotheses  **Text excerpt**: In this study, we pursued a Bayesian meta-analysis to compare the cure rate (CR) and the egg reduction rate (ERR) of different treatments against hookworm infection  considering the diagnostic error of the Kato-Katz thick smear technique. We modeled  diagnostic sensitivity as a function of the infection intensity using the basic model formulation of Bärenbold et al. (2017) [5]. | p. 5 |
|  | **METHODS** |  |  |  |
|  | *Study design* | **5** | Whether data collection was planned before the index test and reference standard  were performed (prospective study) or after (retrospective study)  This is a retrospective study.  **Text excerpt**: The studies from which the data used in this analysis have been obtained have been published elsewhere [6, 7-11]. | p. 5 |
|  | *Participants* | **6** | Eligibility criteria  **Text excerpt**: The studies from which the data used in this analysis have been obtained have been published elsewhere [6, 7-11]. Details on ethical approvals, trial registration, study  design, informed consent procedures, potential risks and benefits are provided in the aforementioned studies. | p. 5 |
|  |  | **7** | On what basis potentially eligible participants were identified  (such as symptoms, results from previous tests, inclusion in registry)  **Text excerpt**: The studies from which the data used in this analysis have been obtained have been published elsewhere [6, 7-11]. Details on ethical approvals, trial registration, study design, informed consent procedures, potential risks and benefits are provided in the aforementioned studies. | p. 5 |
|  |  | **8** | Where and when potentially eligible participants were identified (setting, location and dates)  **Text excerpts**: - The studies from which the data used in this analysis have been obtained have been published elsewhere [6, 7-11]. Details on ethical approvals, trial registration, study design, informed consent procedures, potential risks and benefits are provided in the aforementioned studies.  -We analyzed data from six randomized trials in Côte d'Ivoire, Lao People's Democratic Republic, and Tanzania, which assessed the efficacy and safety of different treatments against STH infection [6, 7-11]. | p. 5 |
|  |  | **9** | Whether participants formed a consecutive, random or convenience series  **Text excerpt**: The studies from which the data used in this analysis have been obtained have been published elsewhere [6, 7-11]. Details on ethical approvals, trial registration, study  design, informed consent procedures, potential risks and benefits are provided in the aforementioned studies. | p. 5 |
|  | *Test methods* | **10a** | Index test, in sufficient detail to allow replication  **Text excerpt**: The studies from which the data used in this analysis have been obtained have been published elsewhere [6, 7-11]. Details on ethical approvals, trial registration, study  design, informed consent procedures, potential risks and benefits are provided in the aforementioned studies. | p. 5 |
|  |  | **10b** | Reference standard, in sufficient detail to allow replication |  |
|  |  | **11** | Rationale for choosing the reference standard (if alternatives exist) |  |
|  |  | **12a** | Definition of and rationale for test positivity cut-offs or result categories  of the index test, distinguishing pre-specified from exploratory  **Text excerpt**: The studies from which the data used in this analysis have been obtained have been published elsewhere [6, 7-11]. Details on ethical approvals, trial registration, study  design, informed consent procedures, potential risks and benefits are provided in the aforementioned studies. | p. 5 |
|  |  | **12b** | Definition of and rationale for test positivity cut-offs or result categories  of the reference standard, distinguishing pre-specified from exploratory |  |
|  |  | **13a** | Whether clinical information and reference standard results were available  to the performers/readers of the index test  **Text excerpt**: The studies from which the data used in this analysis have been obtained have been published elsewhere [6, 7-11]. Details on ethical approvals, trial registration, study  design, informed consent procedures, potential risks and benefits are provided in the aforementioned studies. | p. 5 |
|  |  | **13b** | Whether clinical information and index test results were available  to the assessors of the reference standard |  |
|  | *Analysis* | **14** | Methods for estimating or comparing measures of diagnostic accuracy  **Text excerpt**: Posterior samples of sigma_(t)^d)^2 and k were used to obtain the posterior distribution of the sensitivity as a function of infection intensity via the relationship  We simulated data for mean infection intensities of 0-500 EPG for either one, two, or  four Kato-Katz thick smears. For one and two Kato-Katz thick smears it was assumed  that the replicate samples were analyzed on the same day, in the case of four Kato-Katz  thick smears it was assumed that two samples were analyzed on one day. | p. 10 |
|  |  | **15** | How indeterminate index test or reference standard results were handled  **Text excerpt**: All individuals were included in this analysis as for missing or indeterminate values missing at random (MAR) can be assumed. | p. 9 |
|  |  | **16** | How missing data on the index test and reference standard were handled  **Text excerpt**: All individuals were included in this analysis as for missing or indeterminate values missing at random (MAR) can be assumed. | p. 9 |
|  |  | **17** | Any analyses of variability in diagnostic accuracy, distinguishing pre-specified from exploratory  **Text excerpt**: Posterior samples of sigma_(t)^d)^2 and k were used to obtain the posterior distribution of the sensitivity as a function of infection intensity via the relationship  We simulated data for mean infection intensities of 0-500 EPG for either one, two, or  four Kato-Katz thick smears. For one and two Kato-Katz thick smears it was assumed  that the replicate samples were analyzed on the same day, in the case of four Kato-Katz  thick smears it was assumed that two samples were analyzed on one day. | p. 10 |
|  |  | **18** | Intended sample size and how it was determined  **Text excerpt**: The studies from which the data used in this analysis have been obtained have been published elsewhere [6, 7-11]. Details on ethical approvals, trial registration, study  design, informed consent procedures, potential risks and benefits are provided in the  aforementioned studies. | p. 5 |
|  | **RESULTS** |  |  |  |
|  | *Participants* | **19** | Flow of participants, using a diagram  **Text excerpt**: The studies from which the data used in this analysis have been obtained have been published elsewhere [6, 7-11]. Details on ethical approvals, trial registration, study  design, informed consent procedures, potential risks and benefits are provided in the  aforementioned studies. | p. 5 |
|  |  | **20** | Baseline demographic and clinical characteristics of participants  **Text excerpt**: Table 1 shows the mean hookworm infection intensities at baseline in the included studies from Côte d'Ivoire, Lao People's Democratic Republic, and Tanzania. All of the  infections were classified as light infections (i.e. <2,000 EPG [12]. The sample were similar for the different treatment arms within the individual studies, with exception of the trial in Lao People's Democratic Republic where approximately twice as many children were assigned to two treatment arms (albendazole plus oxantel and albendazole plus oxantel plus pyrantel) [9]. There were slight differences in CRs and ERRs for the same treatments. For instance, there were three trials administering 500 mg mebendazole with CRs of 13%, 18%, and 24% and ERRs of 53%, 45%, and 11%, respectively [9-11]. | p. 10 |
|  |  | **21a** | Distribution of severity of disease in those with the target condition  **Text excerpt**: Table 1 shows the mean hookworm infection intensities at baseline in the included studies from Côte d'Ivoire, Lao People's Democratic Republic, and Tanzania. All of the  infections were classified as light infections (i.e. <2,000 EPG [12]. | p. 10 |
|  |  | **21b** | Distribution of alternative diagnoses in those without the target condition |  |
|  |  | **22** | Time interval and any clinical interventions between index test and reference standard  **Text excerpt**: For each individual, two stool specimens were collected over two consecutive days at baseline and treatment follow-up, usually 14-21 days post-treatment, while two readings were made per specimen. All slides were read within 1 hour after preparation to avoid degeneration of hookworm eggs on microscope slides. | p. 5, 6 |
|  | *Test results* | **23** | Cross tabulation of the index test results (or their distribution)  by the results of the reference standard  Table 2 and 3. | p. 11, 12 |
|  |  | **24** | Estimates of diagnostic accuracy and their precision (such as 95% confidence intervals)  **Text excerpt**: The estimate of the day-to-day variation in egg excretion was 1.18 (95% BCI 1.14-1.23) and of the egg aggregation parameter was 9.80 (95% BCI 9.00-10.65). Estimates of the sensitivity of the Kato-Katz thick smear technique are shown in Fig 2. For `true'  intensities of an individual with a hookworm infection above 50 EPG the sensitivity was  above 90% for four Kato-Katz thick smears obtained from two stool specimens. For two  Kato-Katz thick smears the sensitivity dropped to between 72% and 82 % and in the  case of only a single Kato-Katz thick smear it was as little as between 55% and 67%.  For hookworm infection intensities of more than 350 EPG, the sensitivity was above  90% irrespective of the number of Kato-Katz thick smears examined. | p. 13 |
|  |  | **25** | Any adverse events from performing the index test or the reference standard |  |
|  | **DISCUSSION** |  |  |  |
|  |  | **26** | Study limitations, including sources of potential bias, statistical uncertainty, and generalisability  **Text excerpt**: Our study has several limitations. First, we addressed uncertainty by linking the  mean infection intensity at follow-up with the aggregation of the worms to improve  model fitting. The aggregation parameter of the worms in the population is estimated  well but with considerable uncertainty, although we linked the aggregation parameter to  the mean infection intensity and the prevalence [13]. Furthermore, there were treatment  arms where the estimates of the ERR have a rather large uncertainty compared to the  estimates of the mean infection intensity at baseline and follow-up. This is the case for  low ERRs. Due to the limited number of trials per treatment, we were unable to include  a random effect to account for the variation between the trials [14]. | p. 16 |
|  |  | **27** | Implications for practice, including the intended use and clinical role of the index test  **Text excerpt**: Our results show that the diagnostic sensitivity increases with the sampling effort. Nevertheless, WHO recommends to collect only one stool sample to be subjected to a  single Kato-Katz thick smear, which can have implications as the CR and ERR are underestimated. | p. 15, 16 |
|  | **OTHER INFORMATION** |  |  |  |
|  |  | **28** | Registration number and name of registry  **Text excerpt**: The studies from which the data used in this analysis have been obtained have been published elsewhere [6, 7-11]. Details on ethical approvals, trial registration, study  design, informed consent procedures, potential risks and benefits are provided in the  aforementioned studies. | p. 5 |
|  |  | **29** | Where the full study protocol can be accessed  **Text excerpt**: The studies from which the data used in this analysis have been obtained have been published elsewhere [6, 7-11]. Details on ethical approvals, trial registration, study  design, informed consent procedures, potential risks and benefits are provided in the  aforementioned studies. | p. 5 |
|  |  | **30** | Sources of funding and other support; role of funders |  |
|  |  |  |  |  |

# References

1. Keiser J, Utzinger J. Efficacy of current drugs against soil-transmitted helminth

infections: systematic review and meta-analysis. JAMA. 2008;299: 1937-1948.

doi:10.1001/jama.299.16.1937.

1. Moser W, Schindler C, Keiser J. Drug combinations against soil-transmitted

helminth infections. Adv in Parasitol. 2019;103: 91-115.

1. Clarke NE, Doi SAR, Wangdi K, Chen Y, Clements ACA, Nery SV. Efficacy of

anthelminthic drugs and drug combinations against soil-transmitted helminths: a

systematic review and network meta-analysis. Clin Infect Dis. 2019;68: 96–105.

doi: 10.1093/cid/ciy423.

1. Moser W, Bärenbold O, Mirams GJ, Cools P, Vlaminck J, Ali SM, et al.

Diagnostic comparison between FECPA2 and the Kato-Katz method for

analyzing soil-transmitted helminth eggs in stool. PLoS Negl Trop Dis.

2018;12:e0006562. doi:10.1371/journal.pntd.0006562.

1. Bärenbold O, Raso G, Coulibaly JT, N’Goran EK, Utzinger J, Vounatsou P.

Estimating sensitivity of the Kato-Katz technique for the diagnosis of

Schistosoma mansoni and hookworm in relation to infection intensity. PLoS Negl

Trop Dis. 2017;11:e0005953. doi:10.1371/journal.pntd.0005953.

1. WHO. Guideline: preventive chemotherapy to control soil-transmitted helminth

infections in at-risk population groups. Geneva: World Health Organization; 2017.

1. Moser W, Coulibaly JT, Ali SM, Ame SM, Amour AK, Yapi RB, et al. Efficacy

and safety of tribendimidine, tribendimidine plus ivermectin, tribendimidine plus

oxantel pamoate, and albendazole plus oxantel pamoate against hookworm and

concomitant soil-transmitted helminth infections in Tanzania and Côte d’Ivoire: a

randomised, controlled, single-blinded, non-inferiority trial. Lancet Infect Dis.

2017;17: 1162–1171. doi:10.1016/S1473-3099(17)30487-5.

1. Coulibaly JT, Hiroshige N, N’Gbesso YK, Hattendorf J, Keiser J. Efficacy and

safety of ascending dosages of tribendimidine against hookworm infections in

children: a randomized controlled trial. Clin Infect Dis. 2019;69: 845–852.

doi:10.1093/cid/ciy999.

1. Speich B, Ame SM, Ali SM, Alles R, Huwyler J, Hattendorf J, et al. Oxantel

pamoate-albendazole for Trichuris trichiura infection. N Engl J Med. 2014;370:

610–620. doi:10.1056/NEJMoa1301956.

1. Speich B, Ali SM, Ame SM, Bogoch II, Alles R, Huwyler J, et al. Efficacy and

safety of albendazole plus ivermectin, albendazole plus mebendazole, albendazole

plus oxantel pamoate, and mebendazole alone against Trichuris trichiura and

concomitant soil-transmitted helminth infections: a four-arm, randomised

controlled trial. Lancet Infect Dis. 2015;15: 277–284.

doi:10.1016/S1473-3099(14)71050-3.

1. Palmeirim MS, Ame SM, Ali SM, Hattendorf J, Keiser J. Efficacy and safety of a

single dose versus a multiple dose regimen of mebendazole against hookworm

infections in children: a randomised, double-blind trial. EClinicalMedicine.

2018;1: 7-13.

1. Levecke B, Anderson RM, Berkvens D, Charlier J, Devleesschauwer B,

Speybroeck N, et al. Mathematical inference on helminth egg counts in stool and

its applications in mass drug administration programmes to control

soil-transmitted helminthiasis in public health. Adv Parasitol. 2015;87: 193-247.

1. Benjamin-Chung J, Pilotte N, Ercumen A, Grant JR, Maasch JR, Gonzalez AM,

et al. Comparison of multi-parallel qPCR and double-slide Kato-Katz for

detection of soil-transmitted helminth infection among children in rural

Bangladesh. PLoS Negl Trop Dis. 2020;14, e0008087.

1. Eshetu T, Aemero M, Zeleke AJ. Efficacy of a single dose versus a multiple dose

regimen of mebendazole against hookworm infections among school children: a

randomized open-label trial. BMC Infect Dis. 2020;20:376.

doi:10.1186/s12879-020-05097-1.
